# Why woody plant modularity through time and space must be integrated in fire research?

**DOI:** 10.1093/aobpla/plad029

**Published:** 2023-05-23

**Authors:** Marco Antonio Chiminazzo, Tristan Charles-Dominique, Davi Rodrigo Rossatto, Aline Bertolosi Bombo, Alessandra Fidelis

**Affiliations:** Lab of Vegetation Ecology, Instituto de Biociências, Universidade Estadual Paulista (UNESP), Avenida 24-A, 1515, Rio Claro 13506-900, Brazil; AMAP, Université Montpellier, CIRAD, CNRS, INRAE, IRD, Montpellier, France; Institute of Ecology and Environmental Sciences, Paris CNRS UMR 7618, Sorbonne University, Paris, France; Departamento de Biologia, Faculdade de Ciências Agrárias e Veterinárias, Univ. Estadual Paulista ‘Júlio de Mesquita Filho’, UNESP Campus de Jaboticabal, Jaboticabal, SP, CEP 14884-000, Brazil; Lab of Vegetation Ecology, Instituto de Biociências, Universidade Estadual Paulista (UNESP), Avenida 24-A, 1515, Rio Claro 13506-900, Brazil; Lab of Vegetation Ecology, Instituto de Biociências, Universidade Estadual Paulista (UNESP), Avenida 24-A, 1515, Rio Claro 13506-900, Brazil

**Keywords:** Climate change, fire, grassy biomes, growth form, *Miconia albicans*, plant architecture, resprouting, savanna, shrub, tree

## Abstract

Different ecosystems evolved and are maintained by fire, with their vegetation hosting species with a wide diversity of persistence strategies allowing them to insulate their body and resprout new branches after fire disturbance. Changes in fire regime are predicted due to climate change, either by promoting more frequent and/or severe fires or by reducing the number of fire events due to the limitation of fuel load. Predicting the future of fire-driven ecosystems is a complex task as species’ survival depends on many factors that vary in space and time. Since plants are constantly experiencing new environments as they grow through meristem development, woody plant modularity, modules morpho-physiological aspects and their integration should be considered when investigating species strategies in fire-prone ecosystems: according to their position and their tissue composition, plants’ modules experience fire differently and will contribute differently to other modules and the whole plant survival, with consequences cascading over the overall vegetation structure. Growth modules may hold the key to understanding how fast plants can get protected from fire, ultimately helping us to predict which species will persist across changing fire regimes. We present an empirical example showing how different fire-return intervals translate into distinct pressures on the timing, protection and location of modules, and discuss how these can translate into modifications in the vegetation structure due to climate change.

## Teaser

The response of woody plants to fire is influenced by the timing and spatial distribution of their growth modules relative to the flames. Predictions suggest changes in fire regimes for different vegetation types, emphasizing the need to understand which plant species will thrive and the underlying mechanisms enabling their survival. Considering the modularity of plants over time and space is essential. As plants develop through growth modules' production, their modules can gain increased protection against flames. Additionally, new modules produced farther from the direct impact of the flames may survive by being exposed only to the flame plume. Importantly, the survival capacity of modules during a fire is strongly dependent on trait expression (such as bark production and bud protection) and heat transference from one module to another.

The recognition that plants are composed of different parts has been present in the field of botany since its earliest days (Arber 1950; White 1979). How these parts are organized has been the subject of several studies, aiding botanists and ecologists in understanding how plants cope with their environment through their development (Barthélémy and Caraglio 2007; Klimešová *et al.* 2019). This is because plants are not static organisms, and although being sessile, they are constantly facing different environments as they grow (White 1979; Diggle 1994, 2002). Growing as a modular organism allows plants to balance their development with ecosystem constraints: plants are capable of responding to disturbances and stresses by jeopardizing some modules (and some of these meristems) instead of their whole body (Klimešová *et al.* 2019). This is the case for several species from fire-prone ecosystems, whose development focuses on protecting their meristems from fire and being capable of developing new modules after the disturbance (a strategy known as resprouting; Bond and Midgley 2001). Consequently, resprouting is a widespread strategy across several fire-prone ecosystems (Pausas *et al.* 2016).

Fire-related plant strategies are closely related to each fire-adapted species’ life history and changes across ecosystems with different fire regimes—that is, fire’s size, frequency, intensity, season and extent (Bond and Keeley 2005; Simon *et al.* 2009; Bradstock 2010; Crisp *et al.* 2011; Keeley *et al.* 2011; Dantas and Pausas 2013; Archibald *et al.* 2018). Several drivers were shown to correlate alone to fire regime properties (often grouped in categories such as fuel, moisture, fire ignition and suppression; Bistinas *et al.* 2014; Forkel *et al.* 2017), but these could also correlate with one another at different levels—thus imposing variable outcomes in terms of fire regime prediction (Archibald *et al.* 2018; Kelley *et al.* 2019). Human activities also influence fire regimes, leading to either increases or suppression of fire events, which could lead to changes in fire severity and intensity (Le Page *et al.* 2010; Andela *et al.* 2017; Fréjaville and Curt 2017). Fire regimes are very sensitive to both climate and land management, and their changes may greatly influence the ecology of most species across the globe. Therefore, there is an urgency to predict how species will react to these changes and how this will ultimately cascade into ecological shifts (Archibald *et al.* 2018; Kelley *et al.* 2019; Rogers *et al.* 2020).

Among the many fire-prone ecosystems on earth, tropical grassy biomes have been gaining attention from both the scientific community and decision-makers since they encompass a wide range of vegetation types, from open grasslands to woody savannas (Parr *et al*. 2014). They cover more than 25 % of the planet’s surface (Strömberg and Staver 2022) and provide several ecosystem services (Veldman *et al*. 2015; Lehmann and Parr 2016; Bond 2021). Since these ecosystems are mostly dominated by flammable grasses that fuel surface fires, together they account for 70 % of the planet’s burned area yearly (Archibald *et al.* 2018). Intriguingly, even with such harsh conditions created by these flammable grasses, woody species can thrive in grassy biomes (i.e. savannas) through a variety of growth forms—from small shrubs to large trees. While shrubs and herbaceous layer species allocate most of their biomass belowground, being insulated from fire (Pausas *et al.* 2018), tree species allocate a large part of their biomass aboveground, thus being exposed to the heat generated by the fire (Michaletz and Johnson 2007; Scheffer *et al.* 2014; Bär *et al.* 2019). These allocation strategies of the different growth forms have direct consequences on how exposed their modules are to the heat flux. Consequently, predicting how species with different growth forms are impacted by changes in fire regimes requires an explicit analysis of their structures, physiological mechanisms and an acknowledgement of how the protection over their aerial structures that are directly exposed to the fire translates into survival.

In the frequently burned savannas, the woody vegetation is usually primarily composed of sub-shrubs and forbs capable of storing most of their biomass belowground (Hoffmann and Moreira 2002). Trees and treelets (i.e. short-statured single-stemmed plants) also occur in these environments, although being less abundant (Coutinho 1978). The greater proportion of short-statured plants is the consequence of a phenomenon known as ‘firetrap’ (Bond and van Wilgen 1996). For many species, their survival in savannas is dependent on their capacity to transition from seedlings to young adults capable of displaying their canopy above the flames and exposed only to the fire plume. However, due to frequent fires, species are often top killed or filtered out before achieving a safer height (Bond and Midgley 2001; Grady and Hoffmann 2012; Hoffmann *et al.* 2012). Since saplings from many species are not capable of protecting their vital tissues to survive fire (Grady and Hoffmann 2012), they often develop into young adults only after facing a long fire-free interval between two fire events (Hoffmann *et al.* 2020). Alternatively, those completing their whole life cycle without being able to position their canopy above the flame height due to developmental constraints display morphological, anatomical and physiological strategies allowing them to protect themselves from damages caused by fire (Klimešová and Klimeš 2007; Dantas and Pausas 2013; Dantas *et al.* 2013; Chiminazzo *et al.* 2021). Once trees establish their new modules above the flames (so leaves, buds and thin-barked branches become exposed only to the fire plume), they usually suffer lower mortality from fire (Gignoux *et al.* 1997; Bond and Midgley 2001; Hoffmann *et al.* 2020; Scalon *et al.* 2020; Chiminazzo *et al.* 2021).

Woody species can persist in fire-prone vegetation types through a variety of strategies, but how they will respond to changing fire regimes remains unclear. Climate change affects some of the core climatic variables affecting fire regimes and should—in most parts of the world—create conditions for novel fire regimes. Future scenarios predict for different regions increases in the number of fire events as a response to extreme weather or longer fire intervals due to fuel limitation (Moritz *et al.* 2012; Kelley *et al.* 2019; Rogers *et al.* 2020). Consequently, plants from different vegetation types will be exposed to altered fire regimes to some extent. Given the strong effects of fires on biodiversity, ecosystem functioning and services (Pausas and Keeley 2019), we urgently need to understand (i) which morphological and physiological features enable species to survive in the current and future fire regimes and (ii) how species that could be promoted through changing fire regimes will affect ecosystems. Here, we suggest that understanding how woody species are organized in terms of modularity and where certain functional syndromes are critically required within their modular structure is crucial to address these questions, as woody plant survival has strong implications over fire-prone vegetation structure and functioning.

## Modules Are Set in Different Environments: Their Location and Timing of Development Influence How They Experience Fire

Woody plants develop through repeating developmental units whose properties are shaped by both endogenous and exogenous processes and constraints (Barthélémy and Caraglio 2007). During ontogeny, each newly developed plant part is set in a different environment from the previous parts of the canopy (Diggle 2002). For instance, in forest understories, the modules produced at the beginning of the life of large trees must maintain a high surface/biomass ratio to be able to photosynthesize in the deep shade (Charles-Dominique *et al.* 2018), while modules produced at the canopy levels are exposed to full sunlight (i.e. a different environment from the ones produced in the understory). The environmental constraints to overcome by successive modules along ontogeny are therefore markedly different, making necessary the understanding of (i) which are the critical life stages to overcome for modular plants, (ii) how plants alleviate the environmental constraints through their modularity and (iii) how future environmental changes are going to affect plants’ different life stages and their fitness.

In fire-prone ecosystems, plant modules face different environmental constraints depending not only on their stage of development, but also on their vertical distribution in relation to the direct impact of flames during a fire (Wigley *et al.* 2020). In the case of savannas, the surface fires are fuelled by a continuous layer of flammable C_4_ grasses. This fire regime leads to higher fire-induced damages to structures closer to the ground and to the grassy layer, as the temperatures during fire events are much higher closer to the ground and decrease sharply with increasing height (Trollope 1978; Frost and Robertson 1985; Rodrigues *et al.* 2021)—thus creating a vertical gradient of heat flux inducing different levels of damage to the plants (Graves *et al.* 2014; Chiminazzo *et al.* 2021). This fire regime imposes different constraints on woody plants than other fire regimes. For example, crown fires reach the canopy of tall trees through ‘ladders’ created by short woody species and burn with very high intensity consuming the aboveground parts of tall trees (Pausas *et al.* 2004; Archibald *et al.* 2018). After a fire, many species can persist and thrive in the environment through resprouting, which can occur in three different ways: (i) from structures positioned belowground in specialized bud-bearing organs (Clarke *et al.* 2013; Pausas *et al.* 2018); (ii) from the plant aerial parts such as the main trunk and well-developed branches and stems (Bellingham and Sparrow 2000; Burrows 2002; Burrows *et al.* 2010); or (iii) from a mix of both belowground and aboveground structures (Clarke *et al.* 2013; Souchie *et al.* 2017; Chiminazzo *et al.* 2021). Therefore, the success of woody species after fire events will depend upon at least four biological parameters: (i) the capacity of their aboveground structures to survive fire; (ii) the ability to maintain a viable bud bank aboveground; (iii) the capacity of the plant to protect fast enough the newly built structures before the next fire event and, in case of being top-killed, (iv) the ability to resprout from specialized belowground bud-bearing organs (Bond and Midgley 2001; VanderWeide and Hartnett 2011; Grady and Hoffmann 2012; Clarke *et al.* 2013; Schafer *et al.* 2015; Charles-Dominique *et al.* 2017; Pausas *et al.* 2018). The relative importance of these parameters for explaining individual survival most likely differs among areas with different fire regimes (Pausas *et al.* 2016). Hence, predicting species’ survival abilities to fire in a changing climate will require a systematic evaluation of these parameters taken over a large range of fire regimes (Wigley *et al.* 2020).

Resprouting from structures positioned at the plant base or at their canopy requires a different set of traits and impacts the post-fire competitive abilities of plants that were top-killed (Clarke *et al.* 2013; Chiminazzo *et al.* 2021). For basal resprouters, the type of underground storage organ and the size of their bud bank are key features determining their resprouting success and further persistence in fire-prone landscapes (Pausas *et al.* 2018). On the other hand, species able to resprout higher up in the canopy (i.e. epicormically) must invest in large stem diameter, thick bark, and be able to protect their buds below the bark layer (Lawes *et al*. 2011a, 2011b; Charles-Dominique *et al.* 2015; Pausas 2015; Burrows and Chisnall 2016; Scalon *et al.* 2020). However, in natural conditions, we do not observe a bimodal distribution separating basal and epicormic resprouting, but rather a gradient of responses across modules exposed or not to the strongest effect of fire, with species expressing more than one resprouting strategy being very common (Souchie *et al.* 2017; Chiminazzo *et al.* 2021).

**Figure 1.**
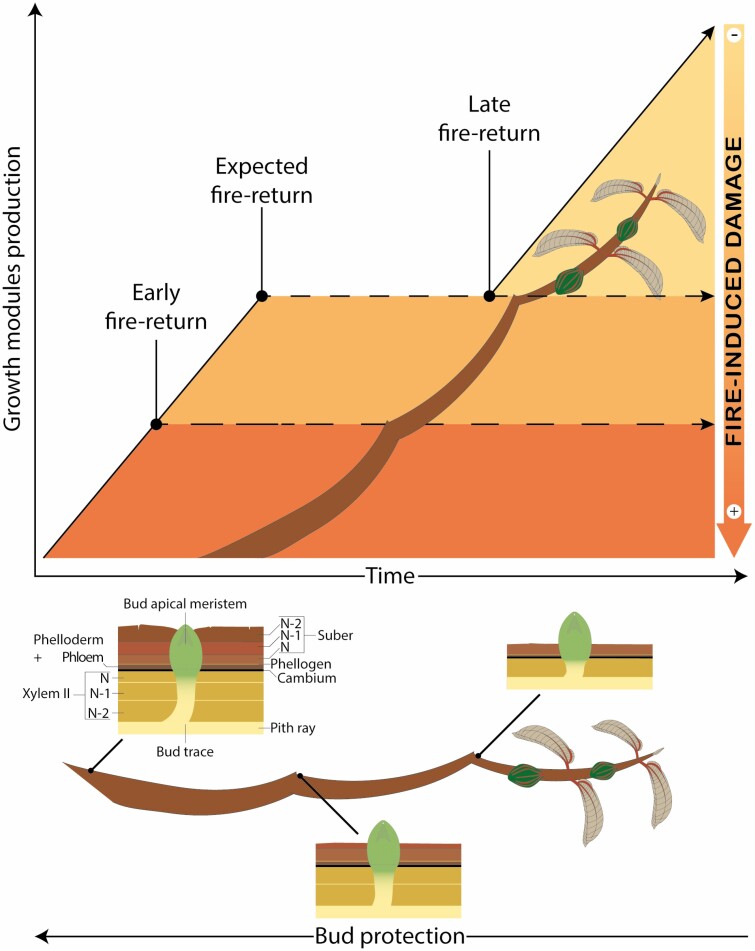
Timing of modules development and their vertical distribution. The setting up of modules in relation to the heat flux influences the post-fire responses of woody species. Note that as plants grow, their growth modules can face different fire-induced damages depending on the fire-return interval and their position in relation to the flames (translating into different heat fluxes over the aboveground structures). While new growth modules and the apical stem meristem can be positioned farther from the ground suffering less fire-induced damage, older modules can gain better protection to their stem and buds as the bark is produced. ‘*N*—number’ indicates the module responsible for the secondary xylem production (xylem II) and suber brands (i.e. suberized, or lignified cells from the phellogen differentiation), with ‘*N*’ indicating the last produced module. Note that the schema shows three modules with the youngest suffering lower impacts of the heat flux in case of late fire return. Although not illustrated here, young modules and their leaves and buds would suffer the highest impacts of the heat flux bottom trapezoid in case of early fire return, with the plant likely being top killed.

Since surface fire induces greater damage to structures positioned closer to the soil (Trollope 1978; Frost and Robertson 1985; Graves *et al.* 2014; Lawes *et al.* 2021), the relative importance of each trait protecting against the heat changes along ontogeny as the plant grows and occupies new spaces (Figure 1). For instance, resprouting from stems and well-developed branches positioned within the flames occur mainly from buds well protected by the bark (Burrows 2002; Charles-Dominique *et al.* 2015; Chiminazzo *et al.* 2021). However, taller structures positioned outside the direct influence of the flames can resprout even from buds unprotected by bark (Chiminazzo *et al.* 2021). As a consequence, the height at which plants resprout affects competition between individuals and species in the post-fire ecosystem, thus influencing the overall vegetation structure after fire (Clarke *et al.* 2013). Hence, predicting vegetation structure in fire-dominated areas cannot be performed without considering how species traits are expressed along the vertical space (Wigley *et al.* 2020).

Growth modules can occupy new spaces by being produced successively and connected to one another (Figure 1). This different vertical distribution exposes each module to different heat fluxes capable of inducing fire damages at different levels, which in turn is also dependent on the module’s tissue conductivity to heat. It is well established that tissue density is directly proportional to tissue conductivity, that is, plant tissues with elevated density have higher capacities to conduce heat (Bauer *et al.* 2010; De Antonio *et al.* 2020). Thus, the degree to which older modules will suffer from first-order effects is highly linked to the thermal conductivity of bark, branch wood and module diameter (Hoffmann *et al.* 2012; Rosell *et al.* 2014; Pausas 2015). Wood and bark density of older modules can also play an important role as they are positioned closer to the grassy layer and may suffer the highest impacts of the flames (first-order effects, Michaletz and Johnson 2007) due to the immediate impact of heat transfer from combusting fuels on plant tissues (Bär *et al.* 2019). If such modules possess higher conductivity (i.e. higher bark and wood density), the heat will successfully be transferred to the top modules, which normally harbour the leaves and are responsible for the production of photosynthates, which maintain older modules and provide the necessary energy to buds’ sprout (Sprugel *et al.* 1991). Additionally, damage to the phloem or to the module cambium can lead to the necrosis of these tissues (Michaletz and Johnson 2007), ultimately blocking the downward translocation of photosynthates to the roots (Bär *et al.* 2019). Underground organs can also suffer from heat transfer, blocking the translocation of reserves necessary for plant resprout after fire (de Moraes *et al.* 2016; Morris 2021). This implies that the plant will suffer from carbon starvation and hydraulic failure (McDowell *et al.* 2018; Bär *et al.* 2019; Partelli-Feltrin *et al.* 2023), just at the moment where the translocation of carbohydrates is important to maintain the modules that survived and to resprout new modules. Hence, these observations highlight the necessity of taking into account specific morpho-physiological traits of modules by considering their production and stage of development in relation to different heat fluxes (Fig. 1): bark is produced around the module conferring higher or lower capacity of heat conductivity to the inner tissues and aerial buds. Complementary, large dormant buds positioned in more-developed modules may also suffer less first-order effects from fires, since they are less susceptible to bud necrosis depending on branch conductivity (Michaletz and Johnson 2006). Therefore, applying growth modules production and their stage of development into models of post-fire plant mortality may help us identify how long it takes for plants to overcome immediate induced damage during fire events.

First-order effects rarely kill mature trees during low- to moderate-intensity fires, mainly because savannas plants possess very well-insulated modules with low heat conductivity (Hoffmann and Solbrig 2003; Balfour and Midgley 2006; Rosell *et al.* 2014; Pausas 2015) but pose a greater risk for young saplings (that do not possess a well-developed or insulated bark; Midgley *et al.* 2010; Lawes *et al.* 2011a; Hoffmann *et al.* 2012, 2020). Even with such a degree of protection, heat transfer can trigger several second-order effects that can be lethal to the plants (Kavanagh *et al*. 2010; Grady and Hoffmann 2012; Bär *et al.* 2019; Hoffmann *et al*. 2020). Second-order effects encompass a wide range of physiological dysfunctions, mainly linked with canopy physiology, and can lead to carbon starvation and general plant hydraulic failure (Bär *et al.* 2019). During fire, the heated plume can drastically increase air temperatures, inducing strong atmospheric vapour pressure deficit (*D*) above the flames and around the plant body (Kavanagh *et al*. 2010), which may lead to embolism once the metastability of the water column within the modules is broken by air seeding (Tyree and Zimmermann 2002). Hence, since fires can induce dysfunctions that disrupt the flow of water from the underground to the leaves, and the flow of carbohydrates from younger modules to old modules and the underground organs, plant modularity must be addressed in fire research by considering modules’ interconnectivity and how the heat flux impacts both young and older modules leading to either death by embolism, necrosis or carbon starvation.

Aside from considering the set-up of modules in space, we need to understand how fire-resisting and -persisting traits are expressed through time and how this timing is compatible with the fire-return interval (Fig. 1). Fire-return intervals are predicted to increase in some areas while decreasing in others (Moritz *et al.* 2012; Kelley *et al.* 2019; Rogers *et al.* 2020). These changes in the frequency of disturbance will most likely promote contrasted survival strategies. In areas subjected to longer fire-return intervals and higher fire intensity, plant species will likely be favoured depending on their ability to slowly develop a strong defence against high-intensity fires by, for example, accumulating bark through low bark shedding (Fig. 1). However, if fire intensity becomes too high, strategies based on basal resprouting with little protection to aboveground structures should be promoted (as seen in Mediterranean ecosystems, Pausas *et al.* 2016). On the other end, in areas subjected to shorter fire-return intervals and hence lower fire intensity and severity, plant species will likely be filtered out according to their ability to resprout and protect their aboveground structures (e.g. by accumulating bark fast, protecting their dormant buds, quickly achieving a large stem diameter) early during their development (Gignoux *et al.* 1997; Shackleton and Scholes 2000; Grady and Hoffmann 2012). Consequently, the success of woody species in both increasing or decreasing fire-frequency scenarios will depend on how they develop between fire events.

## An Example of Fire Impacting Differently the Growth Modules in the Cerrado

**Figure 2.**
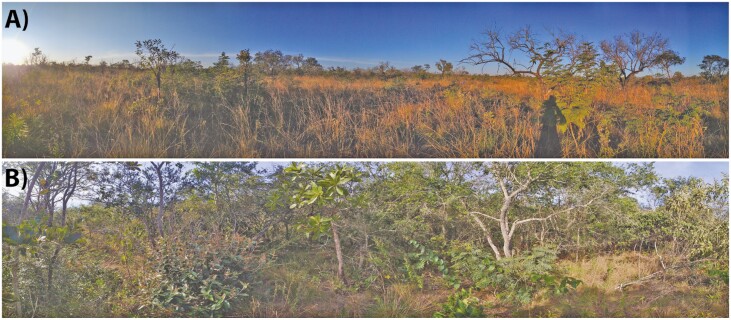
Different types of savanna vegetation found in the Cerrado. (A) Open savanna composed mostly of grasses with few scattered shrubs and sparse trees. (B) Woody savanna with a dense woody cover. Photos were taken at the Santa Bárbara Ecological Station, located in South-Eastern Brazil.

The Cerrado is a tropical savanna region in Brazil, and it is composed of different fire-prone vegetation types. The savanna vegetation can be generally divided into several types according to their canopy closure: from completely open (*campo sujo*) to closed savannas (*cerrado sensu stricto*, Fig. 2), with intermediate levels of woody cover (e.g. *campo cerrado, cerrado ralo*; Ribeiro and Walter 2008). While having distinct structures, these different vegetation types share similar sets of woody species (Coutinho 1978, 1982). Open savannas (*campo sujo*) have a continuous layer of C4 grasses covering ~80% of the vegetation and scattered short trees (up to 4 m); woody savannas (*cerrado sensu stricto*) present less grassy cover (up to ~50%), and a denser woody layer composed of taller trees (up to 7 m tall; Walter 2006; Ribeiro and Walter 2008). The structural differences between these two savannas are due to a tight relationship between the grasses and the woody species and how they impact the ecosystem. As the woody cover increases from the open to the woody savannas, the grassy layer faces a change in species composition and their abundance due to shading (Pilon *et al*. 2021a), diminishing the amount of fuel available for maintaining frequent fires (Miranda *et al.* 2009). Once the woody species composition of these savannas is very similar, species can grow into both vegetation types being exposed to different fire frequencies and canopy closures. This is the case with *Miconia albicans* (Melastomataceae): this species grows both as a tree and a shrub (Goldenberg *et al.* 2022, Fig. 3A and B) through a dichasial sympodial development (Damascos *et al.* 2005; Kaplan 2022) by displaying a greater shoot development during the wet season (Damascos *et al.* 2005; Lenza and Klink 2006).

**Figure 3.**
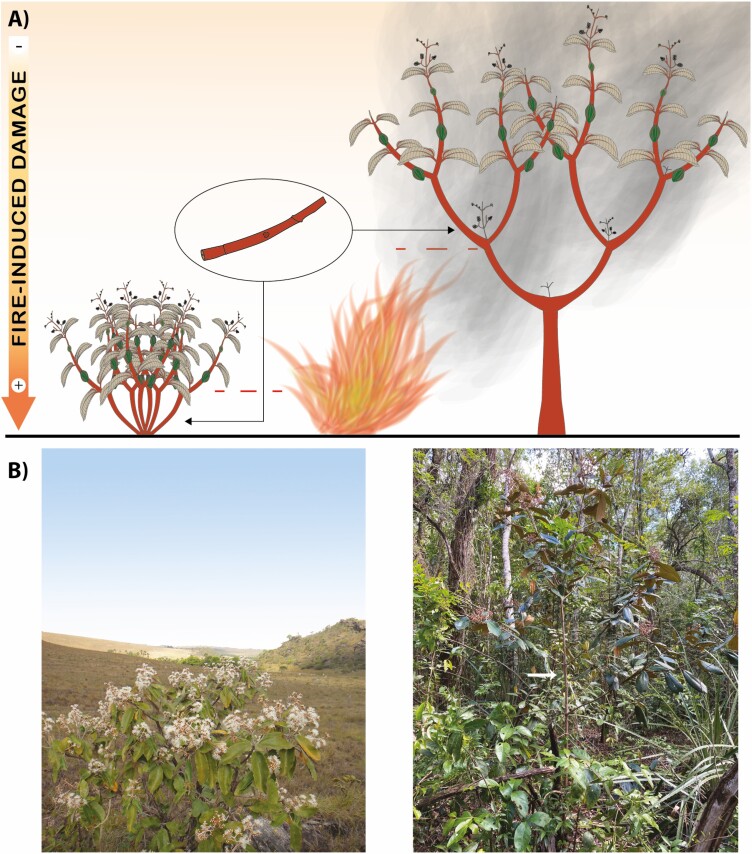
Throughout different growth forms, growth modules are exposed differently to the heat flux during a fire event even though being analogous in terms of development. (A) Schematic illustration showing the structure of the two growth forms of *Miconia albicans*, a widespread Cerrado species. (B) This species can reach its reproductive stage both as a tree (as represented in a young individual growing as a tree in the bottom-right photo) and as a shrub (bottom-left photo). The white arrow in the bottom-right photo indicates *M*. *albicans* main stem. Bottom-left photo by Ana Flávia Alves Versiane.

The aerial buds of *M. albicans* are protected differently when this species grows as a shrub or a tree. When growing in an open savanna as a shrub, its buds are not protected by the bark layer (Chiminazzo *et al.* 2021). However, when growing as a tree in a woody savanna, the buds are well protected (De Antonio *et al.* 2020), being located at the base of depressions in the bark layer (Fig. 1). This difference could be due to either phenotypic plasticity or the stage of development of the sampled growth modules. Charles-Dominique *et al* (2017) found a positive relationship between bark production and aerial bud protection aboveground, meaning that species producing more bark were also able to better protect their buds aboveground. We note here that for most species, the bud location in relation to the bark surface is maintained through time, meaning that buds are growing at the same pace as the bark surface and that their location is actively maintained by a balance between bark production and bud growth (Fig. 1; Chiminazzo *et al.* 2023). The shift in bud protection in *M. albicans*, associated with its development into alternative growth forms, influences its post-fire responses: when growing as a shrub, it resprouts from basal parts after fire events (from the root crown; Pilon *et al*. 2021b); when growing as a tree, it can also resprout from aboveground buds (Chiminazzo and Rossatto, pers. obs.).

**Figure 4.**
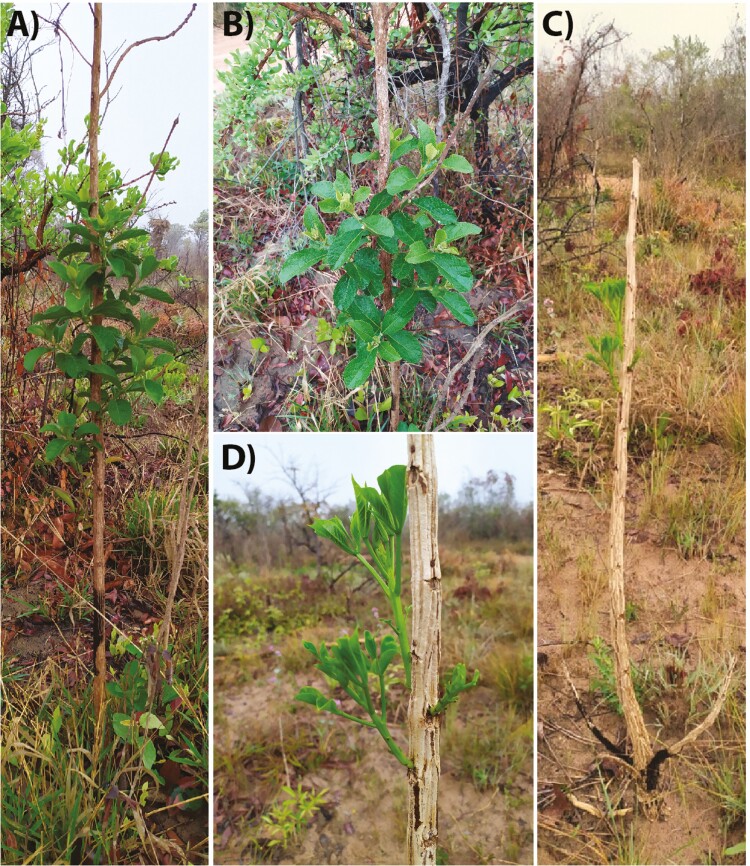
Examples of the vertical distribution of module responses after fire (A, B) *Aegiphila verticillata* (Lamiaceae) resprouting new branches *c.* 1 m aboveground. This species has well-protected aerial buds (buds inserted at depressions in the bark layer, Chiminazzo *et al.* 2021), (C, D) *Handroanthus ochraceus* (Bignoniaceae) shows a low protection by bark (buds exposed above the bark layer, Chiminazzo *et al*., 2021), but it can display aerial resprouting *c.* 1.5 m aboveground. Both *Aegiphila verticillata* and *Handroanthus ochraceus* present belowground bud-bearing organs (woody rhizome), allowing them to persist by resprouting from the belowground (Pilon *et al*. 2021b). Note that aerial resprouting is favoured farther from the ground layer, where fire-induced damage is lower.

When displaying marked seasonal growth, *M. albicans* shows a shoot elongation of ~25 cm per growth season (Damascos *et al.* 2005). The growth rate of *M. albicans* implies that, when growing from seed, it requires 3 years of fire-free interval to position its photosynthesizing structures above the hottest temperatures encountered close to the ground (around 50 cm for open savannas, Rodrigues *et al.* 2021). After resprouting, resources stored as carbohydrates are reallocated to fuel a faster growth reducing the temporal gap needed to reach a height where fire-induced damage is reduced (Hoffmann and Solbrig 2003; Grady and Hoffmann 2012; Hoffmann *et al.* 2012). However, reaching a safe height for the newly developed module is not enough to explain individual survival as modules at the base must resist fire. The survival of modules that are within the flames exposed to the greatest fire-induced damages depends strongly on their diameter (determining their temperature inertia when exposed to fire) and bark production (insulating peripheral tissues) (Fig. 1; Lawes *et al*. 2011a, 2013). Additionally, even out of the greatest effects of fire above the flame zone, buds are exposed to the high temperatures of the fire plume during fire events and still require some level of protection (e.g. trichomes, cataphylls; Chiminazzo *et al.* 2021). If any of the conditions for surviving the aboveground parts are not met, individuals can only persist by rebuilding the entire photosynthesizing structures from belowground buds (Clarke *et al.* 2013). Hence, while the succeeding modules could be morphologically analogous to one another, the important variables to face these three conditions (i.e. shoot elongation in height, growth in diameter, and aerial bud protection) change along the vertical gradient as the modules are being positioned farther from the flames and being exposed to different fire-induced damages through time. These observations extend from *M. albicans* to other species from fire-prone ecosystems, such as *Aegephilla verticillata* (Lamiaceae) and *Handroanthus ochraceus* (Bignoniaceae) (Figs. 1, 3A, and 4). Certainly, these observations are also true for other species, but their commonality remains to be assessed. Surprisingly, plant trait expression and their timing of expression along successive modules have barely been investigated to date (Klimešová *et al*. 2019).

The height at which plant resprouts is highly informative of their ability to tolerate fires of different severity (Fig. 4; Gignoux *et al.* 1997; Hoffmann *et al.* 2009; Midgley *et al.* 2010) and could be an important tool to predict vegetation responses to fire in a future with altered fire regimes together with the expression of traits. These responses are strongly dependent on plant growth form: for woody plants, growing either as a multi-stemmed shrub or a single-stemmed tree affects many important parameters defining plant competition with its neighbours (Götmark *et al.* 2016) and also translates into different exposure to disturbance from the ground up, as it is the case for *M. albicans* (Fig. 3A and B). In woody savannas, the taller and more continuous woody canopy creates a more shaded environment when compared to open savannas (Fig. 2; Carlos and Rossatto 2017; Pilon *et al*. 2021a). Consequently, being able to develop as a tree and positioning its photosynthesizing structures in a higher stratum, outside the direct impacts of the flames, is certainly beneficial, because it allows a better exploration of light availability at the same time it holds off meristematic tissues from the greatest fire damages induced by the heat flux. In areas with frequent fires (e.g. open savannas), allocating resources for the development of single stems aboveground could induce greater risks of large amounts of biomass being exposed to fire damage. Hence, it is easy to observe *M. albicans* developing as a shrub in open savannas and as a short tree in woody savannas (de Antonio *et al*. 2020; Chiminazzo *et al.* 2021; Pilon *et al.* 2021b), with changes in fire-return interval affecting this species’ population growth (Hoffmann 1999).

Finally, there is a need to investigate how plastic protection against fire can be. The strategies of species in fire-prone ecosystems to persist and survive have been identified along the spectrum of either growing in diameter or prioritizing the elongation of shoots—Corky and Lanky strategies (Dantas and Pausas 2013). This strategy could describe the ability of species to survive in areas with fire exclusion (lanky species) or more frequent fires (corky species). However, we still need to evaluate how many species can adjust their strategies by displaying both the ability to resprout after being top-killed, and to develop fast enough to reach a safer height above the flames depending on the fire regime (Gignoux *et al.* 1997; Bond and Midgley 2001). In fire-prone ecosystems, species being able to switch these strategies exist as exemplified here by *M. albicans* and are likely to be favoured under changing fire regimes. Hence, assessing their commonality and distribution across different fire-prone ecosystems can offer important information about how changes in fire regimes will impact different species and vegetation types worldwide.

## Further Steps

Woody species present a wide diversity of traits and strategies allowing them to survive fire, reflecting the high diversity of fire regimes. Therefore, there is a need to investigate in more detail the diversity of fire persistence traits to predict which species will prevail when fire regimes become altered by climate and land uses and why. Thus, we urgently need to study plant responses at the module level, because these responses are sensitive to the modules position relative to the flame height, and they are determined by ontogenetic patterns that translate into plants being protected fast or slow, depending on the ability of plants to cope their development with the environment. Studying fire persistence traits of woody plants and their inclusion in intermediate strategies should be considered priority tasks, since the structure of open ecosystems is strongly affected by changes in woody plant density worldwide, as a result of altered fire regimes.

Further studies should focus on how modules at different stages of development are impacted by various heat fluxes considering their vertical distribution in relation to the flames, and how the surviving modules avoid post-fire death caused by second-order effects such as hydraulic failure and vital inner tissues’ necrosis. This approach offers a promising opportunity for us to understand how long it takes for woody plants to become protected from fire either by positioning their foliage and aerial buds in strata where fire induces non-lethal damages or by becoming insulated against the flames. By doing so, it will be possible to assess which strategies will be more likely to allow species to prevail under changing fire regimes, allowing us to direct conservation efforts to the most vulnerable species to these changes.

## Data Availability

All data used in this manuscript is present in the manuscript.

## Notes

**Plants, Ecosystems and Climate. Chief Editor:** Mary Heskel
